# Reducing Pediatric MRI Cancellations Through Early Anesthesia Assessment: A Retrospective Analysis at a Tertiary Care Center

**DOI:** 10.7759/cureus.89869

**Published:** 2025-08-12

**Authors:** Omar M Kabbani, Hamad Alshammari, Ziad A Alzeer, Ibrahim F Alfarhan, Ahmed H Mahran, Mohamed H Mahmoud, Ahmed Haroun M Mahmoud

**Affiliations:** 1 Anesthesiology, King Abdulaziz Medical City, Riyadh, SAU; 2 Medicine, King Saud Bin Abdulaziz University for Health Sciences College of Medicine, Riyadh, SAU; 3 Anesthesiology, King Saud University Medical City, Riyadh, SAU; 4 Pediatric Anesthesiology, King Abdullah Specialized Children's Hospital, Riyadh, SAU

**Keywords:** anesthesia, cancellation, magnetic resonance imaging, pediatrics, preanesthetic assessment

## Abstract

Background: The use of magnetic resonance imaging (MRI) under anesthesia or moderate sedation has grown in the pediatric age group. Many radiology departments have scheduling problems for pediatric MRIs, as well as on-site cancellations and delays. A contributing factor could be due to the common practice of scheduling pediatric patients for MRIs at the last minute without first consulting with an anesthesiologist. The literature has relatively little information regarding the causes of cancellation of pediatric MRI performed under anesthesia. We predicted that early anesthetic assessment of children might reduce cancellations.

Objective: This study aimed to evaluate the relationship between the reduction of MRI cancellations and the early anesthetic assessment conducted ≥48 hours prior to the MRI.

Methods: This is a retrospective case-control study reviewing MRI cancellations in a tertiary-level specialized children's hospital during the period from March 2017 to December 2018. All pediatric patients who underwent MRI under general anesthesia during this period were included for analysis. The sample population was restricted to patients under 14 years old. MRI cases that were cancelled were compared as a case group with MRI cases that were not cancelled as a control group regarding anesthesia assessment, type, demographics, MRI study characteristics, and medical factors.

Results: The study included a total of 258 children, with 38 cases and 220 controls. The median age of the patients was 52 months with an IQR of 46.5 months for the case cohort and 58 months with an IQR of 48 months for the control cohort. Statistical analysis of the data indicates that preanesthetic assessment two days or more prior to the MRI reduces the risk of cancellations when compared to other risk factors (P=0.020, CI: 0.69-1.652).

Conclusions: Preanesthetic evaluations can reduce MRI rescheduling appointments, additional diagnostic tests, and potential delays in patient care. Operational efficiency can also be improved, along with patient safety and satisfaction. Thus, preanesthetic assessment is vital to reducing MRI cancellations and improving the efficiency of MRI services.

## Introduction

Medical technology has advanced dramatically over the last few decades, and its usage for diagnosis and treatment has increased significantly. Magnetic resonance imaging (MRI) is a rapidly expanding diagnostic medical technology that has gained popularity due to its high-resolution imaging capabilities, three-dimensional reconstruction abilities, and absence of ionizing radiation [1]. Physicians utilize MRI to diagnose, stage, and monitor illnesses [2]. MRI images outperform those produced by computed tomography. However, MRI tests are lengthier, noisier, and more unpleasant [3]. This is especially concerning for young patients, who are rarely able to remain motionless for extended periods of time in a loud, constrained environment during MRI [3]. As a result, pediatric patients are frequently in need of general anesthesia or moderate sedation to decrease the irritation and separation anxiety from their parents and to facilitate the MRI procedure.

To provide safe anesthesia for children undergoing an MRI, a preanesthetic examination is recommended to assess the patient, prevent anesthesia complications, assure safety, and optimize resource utilization [4-5]. Furthermore, the preanesthetic examination enables patients and parents to visit the MRI facility and get oriented, lowering parental anxiety and decreasing the chance of consent refusal. There have been multiple proposed anesthesia protocols in the literature for pediatric patients requiring MRI. However, there have been differences in the outcomes due to multiple factors, including variability in the timing of the preanesthesia assessment and the type of anesthetic medications used [5]. 

With a growing demand for pediatric MRI, a longer waiting list, and full anesthesia slots in the preanesthetic clinic, it has become customary to schedule MRI appointments for pediatric patients without prior anesthesia evaluation. [6]. This causes problems and adverse outcomes, particularly in children with numerous comorbidities. There has been a paucity of studies on early anesthetic evaluation in pediatric MRI settings and its impact on cancellation. The purpose of this study is to look at the value of early anesthetic assessment and how it affects the number of pediatric MRI cancellations at a tertiary-level specialized children's hospital.

## Materials and methods

This retrospective cohort study was conducted at King Abdullah Specialized Children's Hospital, a tertiary care children's hospital in Riyadh, Saudi Arabia. The patients were selected from the database of the pediatric anesthesia department of the hospital after approval from the institutional ethics committee of King Abdullah International Medical Research Center (approval number NRC21R/278/06). Using G Power (Ver. 3.1, Heinrich-Heine-Universität Düsseldorf, Düsseldorf, Germany), the sample size that would achieve a sufficient power level was estimated to be no less than 84. Furthermore, consecutive sampling with matched cohorts was utilized to provide a representative sample for the study population. Quota sampling was utilized to ensure that each study cohort had a common characteristic, that is, cancelled MRIs or cases in which MRIs were done. All patients who underwent MRIs under general anesthesia from March 2017 to December 2018 at the hospital were included for analysis. The sample population was restricted to participants under 14 years old. Early preanesthesia assessment was defined as an anesthesia assessment performed 48 hours or more before MRI [7].

Microsoft Excel 2017 (Microsoft Corp., Redmond, WA) was used for data entry, and the IBM SPSS Statistics software version 25 (IBM Corp., Armonk, NY) for data analysis. The medical records of the patients were reviewed to retrieve relevant information, including age, gender, American Society of Anesthesiologists (ASA) score, preanesthesia assessment, and comorbidities [8]. Categorical variables, including gender and comorbidities, are expressed as frequencies and proportions, while continuous variables, including age, are presented by means and standard deviation. All demographics and clinical characteristics are compared across the study cohorts using a t-test or chi-square test for continuous and categorical variables, respectively. All results are reported in terms of differences in proportions and means, with corresponding 95% CI and P-values. The Mantel-Haenszel method was used for the weighting of the sample sizes of the control and the cases. However, the method was used without continuity corrections to minimize the risk of bias. The method was utilized since the number of observations in the case and control cohorts is unmatched. To investigate subgroup differences that might potentially influence the effect size, the regression model was performed using random effects, that is, the maximum likelihood method. The potential sources of heterogeneity included mean age, gender, comorbidities, and ASA score. Significance was considered at p = 0.05.

## Results

Two hundred fifty-eight children were included in this study. There were 38 cases in the cohort group and 220 in the control group. Mean ages for cases and controls were 52 ± 37 and 56 ± 38 months, respectively. Table 1 summarizes patient characteristics and demographics in both groups. When we compared variables between the two groups, only the preanesthesia assessment conducted over two days before MRI sessions displayed a statistically significant difference (P = 0.020; OR = 0.376; CI = 0.69-1.652), with 76% of cases not undergoing the assessment compared to 56% of controls. MRI types depicted a predominance of CNS-related scans in both cases (88.3%) and controls (83.7%), with a non-significant discrepancy (p = 0.35). Moving to Table 2, reasons for cancellations on the day of MRI highlighted distinct factors contributing to cancellations. Family and patient factors accounted for the majority of cancellations (66%). Lastly, Table 3 presents a binary logistic regression that was conducted to examine the association between various patient-related factors and the likelihood of being in the case group (as opposed to the control group). The predictors included gender, comorbidities, ASA status, age, and whether a preanesthesia assessment was done ≥ 2 days before. Of all variables included in the regression model, only the variable “pre-assessment ≥ 2 days” was found to be statistically significant (P = 0.0008). This suggests that patients who had their pre-assessment more than two days before the procedure were significantly associated with lower cancellation rates. Other variables may have trends worth further exploration in larger or more balanced datasets.

**Table 1:** Patient characteristics (cases and controls) MSK: musculoskeletal; ASA: American Society of Anesthesiologists Categorical variables were compared using the chi-square (χ²) test; Continuous variables (mean ± SD) were compared using Student's t-test; For variables with non-normal distribution (e.g., median and IQR), the non-parametric Mann–Whitney U test was used.

Patient characteristics	Case (n=38)	Control (n=220)	P-value	Test statistic
Male, n (%)	17 (44.7%)	124 (56.4%)	0.125	χ² = 2.33
Age in months, mean ± SD	52 ± 37	56 ± 38	0.539	t = -0.62
Age, median (IQR)	46.5 (22.25–72)	48 (22.25–84)	—	—
ASA, n (%)				
I	2 (5%)	21 (10%)	0.392	χ² = 0.73
II	24 (63%)	140 (64%)	0.954	χ² = 0.00
III	11 (29%)	58 (26%)	0.739	χ² = 0.11
IV	1 (3%)	1 (0%)	0.157	χ² = 2.00
Preanesthesia assessment ≥ 2 days, n (%)			0.020	χ² = 5.41
No	29 (76%)	124 (56%)		
Yes	9 (24%)	96 (44%)		
MRI type, n (%)			0.350	χ² = 0.87
CNS	34 (88.3%)	184 (83.7%)		
Other (shoulder/foot/pelvis)	4 (11.7%)	36 (16.3%)		
Rescheduling category			—	—
Family and patient factors	25 (65.7%)	0		
System factors	9 (23.6%)	0		
Other/not stated	4 (10.5%)	0		

**Table 2:** Reasons for cancellations on the day of MRI‡ Percentage values are relative to the total number of cancellations (n = 38). ‡: Note that more than one factor may have been listed as the reason for cancellation. The totals and percentages may vary since each category has defined criteria for valid and missing data.

Reason for cancellation^†^	Number of cancellations (N=38)	Percentage of cancellations (%)
Family and patient factors	25	66%
Refuse to consent (patient or family)	4	10.5%
Intercurrent illness/underlying illness	12	31.6%
Patient not fasted	4	10.5%
Further work-up necessary, too difficult to intubate	3	8%
Late arrival/ no show	2	5.3%
System factors	9	24%
Necessary results unavailable	2	5.3%
Appropriate physician/primary team unavailable	1	2.6%
No bed available	3	8%
Scheduling issues	3	8%
Other/not stated	4	10%

**Table 3:** Logistic regression results Outcome variable: group (Case = 1, Control = 0); Predictors: gender, comorbidities, American Society of Anesthesiologists (ASA) status, age, preanesthesia assessment ≥ 2 Days; Model type: binary logistic regression

Variable	Coefficient	Standard error	z-value	P-value	Odds ratio	95% CI lower	95% CI upper
Intercept	1.1643	0.9544	1.22	0.2225	3.204	0.494	20.797
C(Gender) (T.Male)	0.5441	0.3804	1.4301	0.1527	1.723	0.817	3.632
Comorbidities	1.0471	0.7621	1.3739	0.1695	2.849	0.64	12.691
ASA	-0.5534	0.331	-1.6716	0.0946	0.575	0.301	1.1
Age	0.0008	0.0049	0.1528	0.8785	1.001	0.991	1.01
Q("Pre_Assesment_2 Days")	1.3716	0.4106	3.3405	0.0008	3.942	1.763	8.815

## Discussion

This study sought to determine whether preanesthetic assessment and evaluation are a key component in decreasing MRI cancellations in health care organizations. In our study, the findings indicate that preanesthetic assessments reduce the risk of MRI cancellations (P = 0.020 (OR) 0.376; 95% (CI) 0.69-1.652). Preanesthetic evaluations are particularly important for improving patient compliance [8]. These evaluations provide psychological preparation both for the parent and the patient by reducing anxiety, explaining the procedure, demonstrating to the children the equipment to be used for anesthesia, and giving hands-on explanations of vascular access devices, monitoring equipment, and facemasks. Also, to give age-appropriate answers to all questions asked by the children and their parents. The psychological aspect of the evaluations is preferable to medication for relieving patient anxiety. Case in point, preanesthetic evaluations have been shown to be more effective than medications [9]. Similarly, retrospective research done in 1996 showed that children who took part in a preparation program five to seven days before their surgery experienced the least anxiety when separated from their parents. On the other hand, those informed one day before surgery were the most anxious [10]. As such, preanesthesia evaluations are necessary for the radiologists and other personnel to tailor the MRI procedures for each patient and to minimize the risk of MRI cancellations or rescheduling. More importantly, preanesthesia evaluations allow for patient examination for the presentation of symptoms of any contradicting medical condition. The evaluations consider family history, preexisting conditions, and an examination based on reported symptoms and diagnoses [11]. Based on the examination, the attending physician at the MRI unit can recommend further examinations with different specialists or allocate a specific time for the MRI [12]. As such, pre-evaluations allow for the assessment of potential risk factors for anesthesia complications and even the intended MRI procedures.

In our study, the main reason for cancellation was due to family and patient-related factors (66%). especially because of intercurrent illness and underlying illness. Many of the quoted reasons for cancellation were also similar to other research done in 2009 and 2015. Which included medically unfit patients, procedures/operations not necessary, patients not fasted, staff unavailable, and unavailability of a bed [2, 13]. In contrast, a study done by Hoffman et al. in 2015 showed that the factor with the largest impact on same-day cancellation of anesthesia-supported studies was due to low neighborhood income quintile [2]. This finding implies that patients with the lowest socioeconomic status are at higher risk of cancellation [2]. This can be interpreted as poverty impacting one’s access to healthcare either by limiting material conditions (e.g., lack of transportation) or by diminishing one’s degree of social engagement and control of one’s life [14]. In addition, preferential access to care for higher-income individuals is considered by some to be less of an issue in a universal insurance healthcare system; however, previous Canadian studies have shown that discrepancies still exist [15-16]. In our research, we did not study the socioeconomic status because our patient population was fully covered in a government hospital setup. In contrast, other factors such as gender, comorbidities, and ASA classification showed no significant impact on cancellation rates, suggesting that these elements are less critical in the context of MRI scheduling (OR 1.083, OR 0.317, OR 1.002, respectively). This goes parallel with other research reporting that the medical complexity of patients had no effect on cancellation [2].

Considering the current high cost of medical services, cancellations and rescheduling of appointments are not a welcome occurrence in hospitals. As such, hospital administrators are tasked with balancing operational efficiency and the utilization of healthcare resources [17]. Standardization in part provides a framework for organization within hospitals, such as the setting up of preanesthetic clinics [18]. As such, preanesthetic assessments are essential from an organizational standpoint to ensure hospital staff coordination and optimization of service delivery [18]. Evidently, preanesthetic evaluations can reduce healthcare costs associated with MRI rescheduling appointments, additional diagnostic tests, and potential delays in patient care. Additionally, operational efficiency can be improved, along with patient safety and satisfaction.

Limitations

Nevertheless, this study's retrospective nature may introduce biases, such as selection bias, as it relies on existing medical records. This could affect the accuracy of the data and the generalizability of the findings. While the study includes 258 pediatric patients, the findings may not be generalizable to broader populations. The single-center approach limits the applicability of the results to other healthcare settings, particularly those with different patient demographics or healthcare systems. Potential confounding variables, including socioeconomic status and comorbidities, were not fully controlled for. These factors could influence both the likelihood of MRI cancellations and the effectiveness of early anesthesia assessments. Addressing these limitations in future studies is essential. Prospective multi-center studies with larger sample sizes and controlled variables are recommended to validate these findings and explore additional factors influencing pediatric MRI cancellations.

## Conclusions

Preanesthetic assessment is vital to reducing MRI cancellations and improving the efficiency of MRI services. By ensuring that patients are properly assessed and prepared for their MRI procedures, healthcare facilities can enhance patient safety, optimize resource utilization, and improve overall operational efficiency. Future research should focus on identifying best practices for preanesthetic assessment in the context of MRI procedures to further enhance patient care and outcomes. Furthermore, future studies should incorporate prospective study designs with representative samples.
